# Adults with Down syndrome display altered entrainment of occipital cortical neurons

**DOI:** 10.1093/braincomms/fcag038

**Published:** 2026-02-06

**Authors:** Liana Chinen, Morgan T Busboom, Jiraros Meejang, Olyvia Kastner, Elizabeth Heinrichs-Graham, Tony W Wilson, Max J Kurz

**Affiliations:** Institute for Human Neuroscience, Boys Town National Research Hospital, Omaha, NE 68010, USA; Institute for Human Neuroscience, Boys Town National Research Hospital, Omaha, NE 68010, USA; Institute for Human Neuroscience, Boys Town National Research Hospital, Omaha, NE 68010, USA; Institute for Human Neuroscience, Boys Town National Research Hospital, Omaha, NE 68010, USA; Institute for Human Neuroscience, Boys Town National Research Hospital, Omaha, NE 68010, USA; Department of Pharmacology and Neuroscience, Creighton University, Omaha, NE 68729, USA; Institute for Human Neuroscience, Boys Town National Research Hospital, Omaha, NE 68010, USA; Department of Pharmacology and Neuroscience, Creighton University, Omaha, NE 68729, USA; Institute for Human Neuroscience, Boys Town National Research Hospital, Omaha, NE 68010, USA; Department of Pharmacology and Neuroscience, Creighton University, Omaha, NE 68729, USA

**Keywords:** vision, magnetoencephalography, neuroimaging, Alzheimer’s disease, dementia

## Abstract

Down syndrome is commonly associated with a trisomy of chromosome 21 that often presents an accelerated aging profile and higher probability of developing Alzheimer’s disease-like symptoms at a relatively early age. However, the physiological changes that may contribute to such symptoms remain poorly understood. To begin to address this knowledge gap, we used magnetoencephalographic neurophysiological imaging to assess the entrainment of occipital cortical neurons to a 15 Hz visual stimulus in a cohort of adults with DS without a dementia diagnosis (*N* = 26; Age = 27.65 ± 9.55 years) and a demographically matched cohort of neurotypical controls (*N* = 22; Age = 30.81 ± 8.02 years). Our results indicated that adults with Down syndrome exhibit substantially weaker entrainment of the occipital cortical neurons and elevated spontaneous activity during the prestimulation baseline period compared with the controls. These results suggest that there are alterations in the integrity of occipital neural populations that may be attributable to an imbalance in local GABAergic activity and/or disruption in cholinergic pathways. These changes may affect the strength of resting cortical rhythms, leading to the elevated spontaneous activity observed here, which has been linked to reductions in the dynamic range of neural populations and impairments in perceptual and cognitive processing. These novel results advance our understanding of the occipital cortical physiology seen in adults with Down syndrome and provide foundational knowledge for the development of biomarkers for the early detection of accelerated aging and cognitive decline in those with Down syndrome.

## Teaser

Chinen *et al*. report that adults with Down syndrome have weaker entrainment of the occipital cortical neurons and have an elevated spontaneous activity relative to controls. The altered strength of the resting cortical rhythms likely impacts the available dynamic range of the neural populations.

## Graphical Abstract

**Graphical Abstract:**
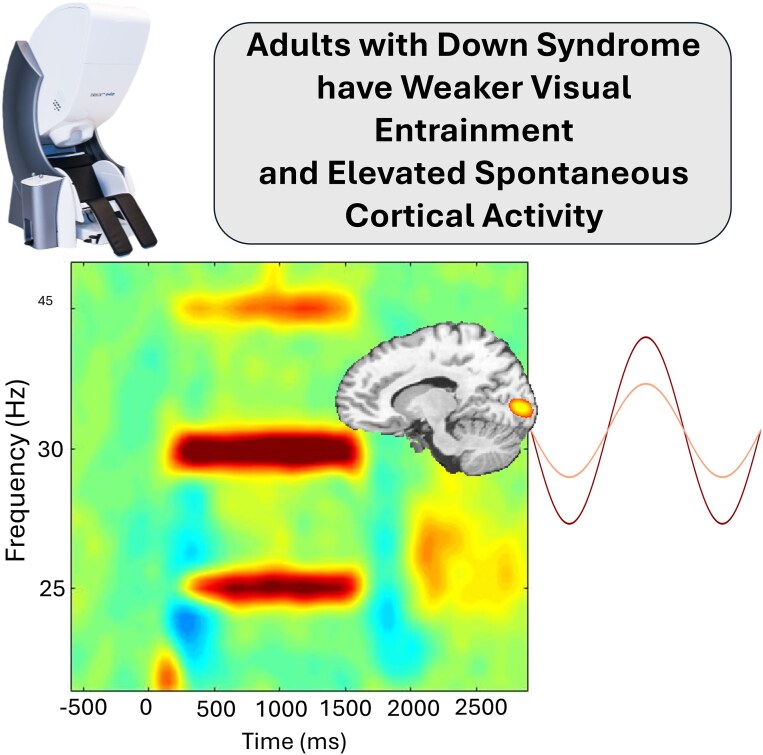

## Introduction

Many individuals with Down syndrome (DS) develop clinical symptoms of Alzheimer’s disease as early as 30 years of age.^1^ With an extra copy of chromosome 21, individuals with DS overexpress the amyloid precursor gene, leading to elevated levels of amyloid-β.^2,3^ Amyloid-β is thought to be the primary driver of dementia in individuals with DS, as it contributes to the formation of amyloid plaques and tau tangles, which have damaging effects on neurons and synaptic connections.^3^ Often, the associated cognitive decline in this patient population is mistaken as part of their intellectual disability or due to comorbidities such as sleep disturbances, seizures and psychiatric conditions that impact cognition.^2,3^ Baseline function is also highly variable among individuals with DS, which presents further challenges to distinguishing dementia like symptoms and deriving accurate diagnoses.^2,4^ All in all, these factors contribute to an underdiagnosis of Alzheimer’s disease (AD) in adults with DS, delaying their access to medications to treat symptoms and limiting the time individuals and families have to plan for the future.^5^ Thus, improved diagnostic tools for AD in individuals with DS are essential.

In recent years, there has been an increasing effort to identify biomarkers for earlier AD diagnoses. While plasma, cerebrospinal fluid and PET neuroimaging biomarkers have shown promise, there is still a need to identify faster, more cost-effective and less invasive paths towards accurate diagnosis.^2^ Along these lines, leveraging noninvasive neuroimaging tools such as MRI, EEG and magnetoencephalography (MEG) has emerged as an area of major interest. Several postmortem and structural neuroimaging studies have revealed that adults with DS have reduced brain volume, shallower sulci, enlarged ventricles and widespread alterations in age-related cortical thinning.^6-10^ In contrast to the largely stable cortical thickness seen in healthy 20- to 30-year-old adults, people with DS appear to have cortical thinning throughout this period, suggesting that adults with DS might be on a pathway for accelerated aging.^9,11^ Additionally, EEG and MEG studies have revealed that individuals with DS have a decreased amplitude and shift in the peak frequency of resting state theta (4–7 Hz) and alpha band (8–12 Hz) cortical oscillations.^12-16^ A few smaller-scale task-based EEG investigations conducted at the electrode level have also noted that the evoked visual potentials for children and adults with DS have longer latencies, diminished response amplitudes and less inhibition with repetitive stimulation.^17-19^ Although these investigations are insightful, the limited neuroimaging landscape strongly suggests that we are still in the early stages of understanding how the genetic alterations associated with DS impacts the brain.

Neuronal entrainment paradigms have been found to be widely useful in identifying and characterizing dysfunction at the cortical level.^20-26^ Visual entrainment occurs when neurons in the occipital cortices synchronize with the frequency of a flickering visual stimulus.^22,27-29^ In the context of AD, the outcomes of these investigations have highlighted that the entrainment of the occipital cortices is stronger in those with AD compared with controls.^21^ In contrast, entrainment generally becomes weaker with increasing age in cognitively healthy controls.^22^ Despite the recently increased use of entrainment paradigms in AD research, no investigation to date has used this methodology in adults with DS. Given that adults with DS have a higher propensity for developing symptoms of AD, characterizing visual entrainment responses in those without a dementia diagnosis may help lay the groundwork for better understanding typical age-related changes seen in those with DS. Furthermore, such data may provide a benchmark for gauging the onset of neural deviations associated with AD and/or other age-related declines seen in those with DS. Thus, the purpose of this study was to use MEG to evaluate whether entrainment of occipital cortical neurons is aberrant in adults with DS. Based on the limited number of visual event-related potential studies with children and adults with DS,^17-19^ we hypothesized that adults with DS would have significantly weaker entrainment within occipital cortices.

## Materials and methods

### Study design, setting and participants

Based on the effect sizes seen in the prior EEG visual event–related potential studies conducted with children and adults with DS^17-19^ (Cohen’s *d* = 1.19), 10 participants per group would have 80% power to detect a similar groupwise difference. A total of 48 participants were included in this cohort design investigation that was conducted at an academic medical institution. Twenty-six had a diagnosis of DS with trisomy of chromosome 21 (age = 27.65 ± 9.55 years, females = 8), and 22 were neurotypical controls without any genetic disorders or major psychiatric or neurological conditions (Age = 30.81 ± 8.02 years, Females = 6). The two groups did not statistically differ by sex or age (*P*  *>* 0.201), indicating that the sample selection bias was reduced. For both groups, participants were excluded from the study if they had metal in the body (e.g. dental braces, metal implants and/or ferromagnetic implants) that would preclude the use of MRI or MEG. The Boys Town National Research Hospital’s Institutional Review Board approved this investigation (IRB #2105XP). Each participant and guardian provided written informed consent and assent following a detailed description of the study. All consenting procedures were obtained according to the Declaration of Helsinki.

### MEG acquisition and experimental paradigm

**Figure 1:**
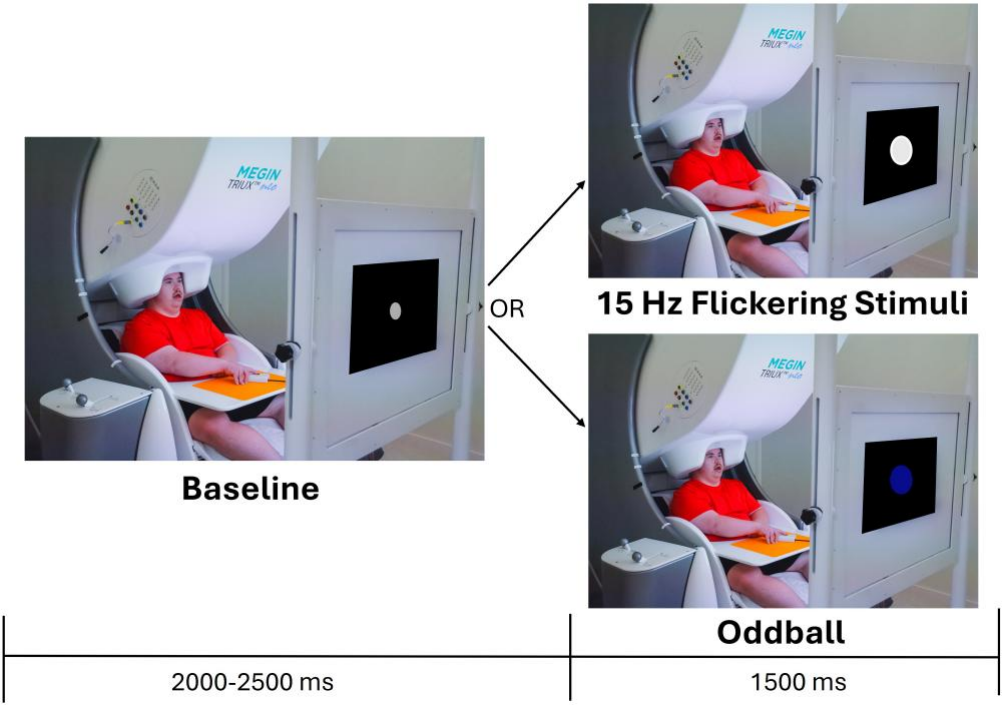
**Experimental design.** Depiction of the experimental paradigm where the participant was seated in the MEG while viewing a flickering stimulus that was presented for 1500 ms. A grey circle was shown during the baseline period that lasted between 2000 and 2500 ms. Intermixed with the trials, a blue dot was shown on the screen and the participant was expected to press a button with their right hand. The oddball trials were used to monitor the participants’ attention to the presented stimuli and were not included in the final analyses. Note that the size of the stimuli on the screen has been increased for visualization purposes. The actual sizes are described in the main text.

All recordings were conducted using a MEGIN Neo system (Helsinki, Finland) with 306 magnetic sensors in a two-layer Vacuumschmelze magnetically shielded room to minimize environmental noise. Neuromagnetic responses were continuously sampled at a rate of 1 kHz with an acquisition bandwidth of 0.1–330 Hz. During the MEG recording, participants sat in a nonmagnetic chair with their head positioned in a helmet housing the MEG sensor array. Participants were instructed to fixate on an entrainment stimulus that flickered at a rate of 15 Hz. The visual stimulus was a small white circle with a 3.5 cm diameter, presented centrally on a black screen 1 metre away. This flickering stimulus was intermixed with odd-ball trials where a blue circle was presented. The duration of the flickering stimulus was 1500 ms, and the interstimulus interval was randomly jittered between 2000 and 2500 ms. A small grey fixation circle with a 0.58 cm diameter was presented during the baseline period. Participants were instructed to press a button when the blue circle appeared to ensure that they were attending to the stimuli and these trials were discarded (Fig. 1). A MEG technologist also continuously monitored participants via real-time audio–video feeds to ensure compliance. Each participant completed 70 entrainment trials, plus the discarded oddballs.

### MRI acquisition and co-registration with MEG

Prior to the MEG recording, five coils were attached to participant’s’ head. These coils, 3 fiducial points, and at least 100 scalp surface points were digitized (FASTRAK, Polhemus Navigator Sciences, Colchester, VT, USA). After participants were positioned in the MEG, an electric current with a unique frequency label (e.g. 322 Hz) was fed to each of the coils, inducing a measurable magnetic field that allowed each coil to be localized in reference to the MEG sensors. Since these coils were also known in head coordinates, we could use them to create a common coordinate system for co-registering each participant’s MEG with their T1-weighted MRI. These high-resolution MPRAGE T1-weighted images were obtained with a Siemens Prisma scanner equipped with a 32-channel head coil using the parameters: TR: 2400 ms; TE: 1.96 ms; flip angle = 8°; FOV: 256 mm; slice thickness: 1 mm slice with no gap; in-plane resolution: 1.0 mm3. Structural images were aligned parallel to the anterior and posterior commissures and transformed into standardized space.

### MEG pre-preprocessing and source imaging

MEG data from each participant was individually corrected for head motion using the MaxFilter software (MEGIN) and subjected to noise reduction using the signal-space separation method with a temporal extension.^30^ All MEG data preprocessing and imaging used the BESA 7.1 software. Cardiac and ocular artefacts (e.g. blinks) were removed using signal space projection (SSP). This artefact correction was accounted for during source reconstruction. The continuous magnetic time series was then divided into 3500 ms epochs (−600 to 2900 ms), with the baseline extending from −600 to 0 ms before the onset of the flickering stimulus. Epochs were rejected per participant using a fixed threshold method, supplemented with visual inspection. To account for variance in neural response amplitudes and distances between the MEG sensor array and the participant’s head, we used an individually determined threshold based on the signal distribution for both signal amplitude and gradient to reject artefacts. After artefact rejection, on average individuals with DS had 59.88 ± 4.02 trials and controls had 61.23 ± 4.10 trials remaining. There was no significant difference in the number of accepted trials per group (*P* = 0.261).

Artefact-free epochs for each sensor were then transformed into the time-frequency domain using complex demodulation with a time–frequency resolution of 1 Hz/50 ms. The resulting spectral power estimations per sensor were averaged across trials, generating time–frequency plots of mean spectral density per participant. The sensor-level data were then normalized per time-frequency bin using the respective bin’s baseline power, which was calculated by averaging the power during the 600 ms baseline period. The specific time–frequency windows used for source imaging were determined by statistical analysis of the sensor-level time–frequency data across all participants and the entire array of gradiometers. Each data point was initially evaluated using a mass univariate approach based on the general linear model. To reduce the risk of false positive results while maintaining reasonable sensitivity, a two-stage procedure was followed to control for Type 1 error. In the first stage, paired-sample *t*-tests were conducted to test for differences from baseline at each data point and the output time series of *t*-values was threshold at *P* < 0.05 to define time–frequency bins containing potentially significant responses across all participants. In stage two, the time–frequency bins that survived the threshold were clustered with temporally and/or spectrally neighbouring bins that were also above the threshold (*P* < 0.05), and a cluster value was derived by summing all of the *t*-values of all data points in the cluster. Nonparametric permutation testing was then used to derive a distribution of cluster-values and the significance level of the observed clusters (from stage one) were tested directly using this distribution (Maris & Oostenveld, 2007). For each comparison, 1000 permutations were computed to build a distribution of cluster values. Based on these analyses, only the time–frequency windows that contained significant responses across all trials were subjected to imaging. Using these time–frequency windows and a pre-stimulus noise period of equal duration and bandwidth, a minimum variance vector beamformer based on the cross-spectral densities was used to calculate the source power across the entire brain volume per participant at a 4.0 × 4.0 × 4.0 mm resolution.^31,32^

The resulting images were grand-averaged, and the virtual sensor time series were extracted from the peak voxels by applying the sensor weighting matrix derived through the forward computation to the preprocessed signal vector.^33,34^ The neural time courses were subsequently used to estimate the signal envelope across the bandwidth used for imaging. The average relative power across the window of interest (i.e. normalized to the baseline) during the active stimulation period was the primary outcome measure, and this was used to assess group differences. Additionally, our secondary outcome measure was the average power across the baseline window and this was used to assess for differences in basal neural activity.

### Statistical analysis

Separate repeated measures ANOVA models (group × harmonic) with age as a covariate of no interest were used to determine if there were group differences in entrainment strength during the stimulation and spontaneous activity during the baseline period. A Bonferroni corrected *post hoc* was used to assess the group by harmonic differences. Cohen’s *d* was also calculated to determine the magnitude of group differences. A Cohen’s *d* of 0.2 was a small effect size, 0.5 was a medium effect size and a 0.8 was considered a large effect size.^35^ All statistical analyses were performed at the 0.05 alpha level in JASP (version 0.19.1.0).

## Results

### Sensor-level analysis and source imaging

**Figure 2:**
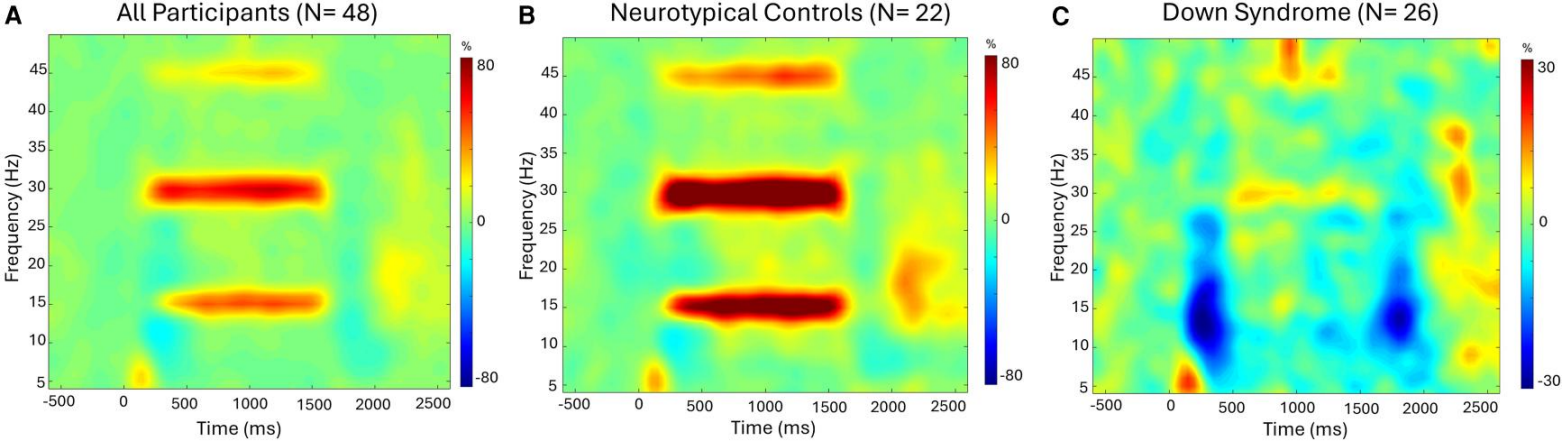
**Sensor level neural responses.** Group-averaged time frequency spectrograms for a sensor near the occipital cortices are shown where the x-axis depicts time (ms) and the y-axis corresponds to frequency. 0 ms is the onset of the 15 Hz flickering stimulus. The respective panels show the spectrograms generated based on the data from all of the participants (**A**), only the neurotypical controls (*N* = 22) (**B**), and the adults with Down syndrome (*N* = 26) (**C**). The grand averaged and control group spectrograms revealed that there was a robust power increase at 15 Hz that stretched across the 250–1600 ms time window, with strong responses also seen at the second and third harmonics (i.e. 30 and 45 Hz, respectively). However, this was not the case for the adults with Down syndrome, where entrainment to the 15 Hz stimulus was absent and responses at the second and third harmonics were very weak. Note that the scale of the spectrogram for the Down syndrome group is shown at a much more sensitive level than the grand-averaged and control spectrograms. This was necessary to illustrate the harmonic responses, although it also makes the power decrease in the 10–20 Hz range following stimulus onset and offset appear more strongly. A color-blind friendly image is shown in the Supplementary material.

Statistical analysis of the time–frequency spectrograms across all participants revealed a robust power increase in the posterior gradiometers at the entrainment frequency (i.e. 15 Hz) and its harmonics (i.e. 30 and 45 Hz). The noted power increase began ∼250 ms after the onset of the stimulus and was sustained until ∼1600 ms (Fig. 2). Permutation testing of the sensor-level data revealed that the stimulus induced a significant 14–16 Hz power increase that reached its peak and was sustained across the 800 to 1400 ms time window (*P* < 0.001, permutation corrected). Furthermore, there were significant power increases at the second and third harmonics, 29–31 Hz and 44–46 Hz, which were also sustained across the 800 to 1400 ms time window (*P* < 0.001, permutation corrected). For enhanced visualization, we subsequently separated the time-frequency spectrograms of the two groups to get a qualitative depiction of the power differences (Fig. 2). Visual inspection of the spectrograms revealed that the controls exhibited a robust entrainment response at the stimulation frequency. On the contrary, increases in 14–16 Hz power during the stimulation were absent in the adults with DS and responses in the respective harmonics were also notably weaker. Finally, there was also a decrease in alpha (8–12 Hz) power that appeared shortly after the onset of the stimulus and was more evident in the spectrogram of the adults with DS, although this was partly due to the absence of entrainment at the fundamental frequency.

**Figure 3:**
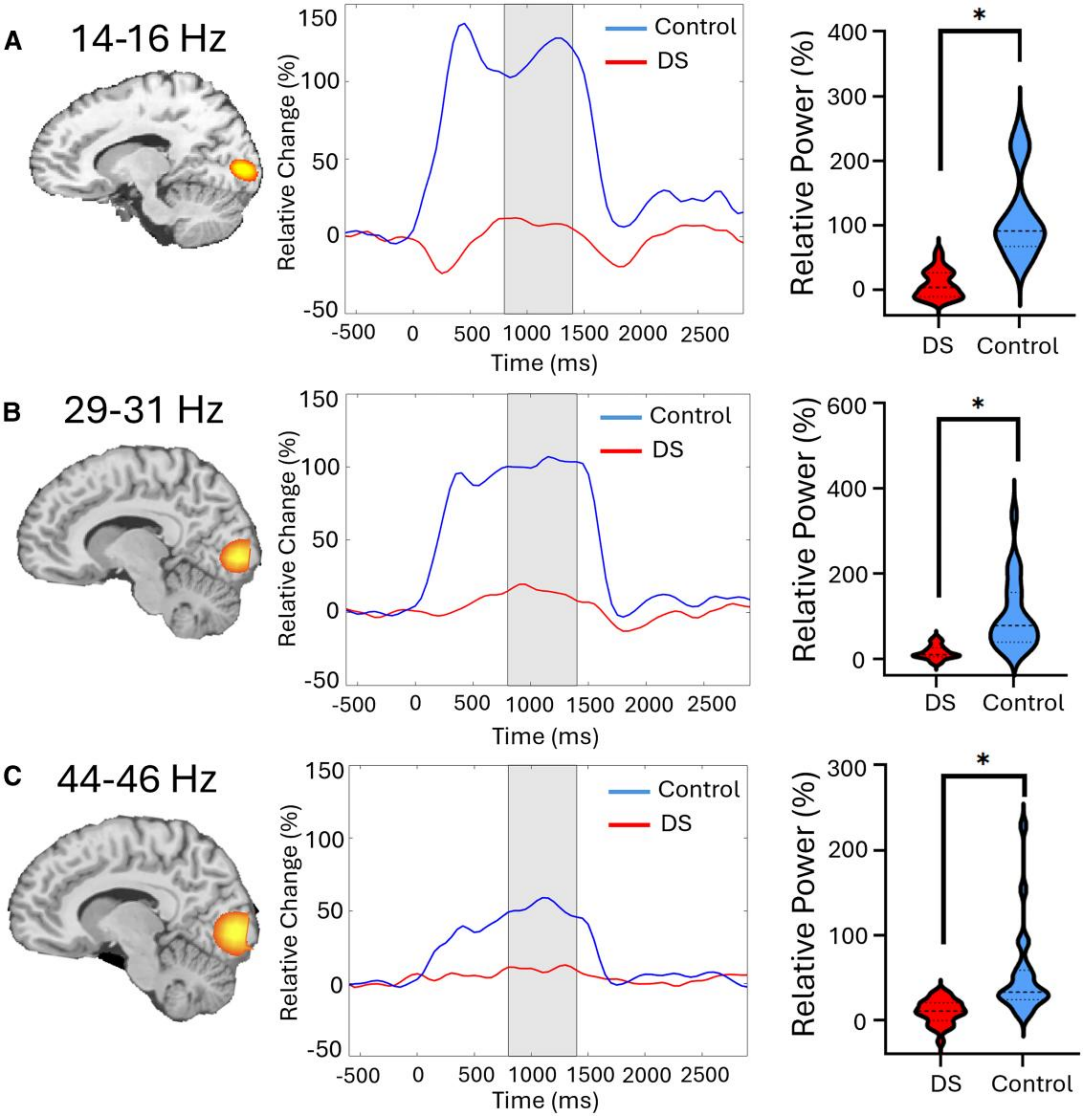
**Relative strength of the entrainment response in occipital cortical neurons.** The left panels display the beamformer images of the entrainment responses across the fundamental (**A**), second harmonic (**B**) and third harmonic (**C**). The middle panels show the neural time series per group that was extracted from the peak voxel of the respective image. The blue line represents the neurotypical controls (*N* = 22), and the red line represents the adults with Down syndrome (DS) (*N* = 26). Time 0 ms reflects the onset of the 15 Hz visual stimulus, while the shaded area represents the time window used for imaging and for computing the mean response strength. The violin plots in the right panel show the average strength of the entrainment response per group, where the median (50th percentile) is indicated by the dashed line and the quartiles (25th and 75th) are marked by the dotted lines. A repeated-measures ANOVA model (group × harmonic) with age as a covariate of no interest was used to evaluate differences in the cortical responses. (**A**) The 14–16 Hz entrainment was significantly [*t*(45) = −7.54; *P* < 0.001] weaker in the adults with DS when compared with the controls. (**B**) The 29–31 Hz harmonic was also weaker [*t*(45) = −5.07; *P* < 0.001] in the adults with DS. (**C**) The 44–46 Hz harmonic was likewise weaker [*t*(45) = −3.73; *P* = 0.008] in the adults with DS. *P < 0.05.

A beamformer was used to image the source of the 14–16 Hz, 29–31 Hz and 44–46 Hz power increases seen across the 800–1400 ms time window in each participant using a −600 to 0 ms baseline. Separately these images were group and grand-averaged, which revealed that the activity emanated from the bilateral occipital cortices for each frequency band (Fig. 3). The neural time courses were subsequently extracted for each participant based on the peak voxel location in the respective grand-averaged images. The neural time courses imply that the occipital cortical activity was notably weaker for the adults with DS. The average response strength per participant was subsequently calculated across the 800–1400 ms active stimulation time window, and the −600 to 0 ms time window for the spontaneous baseline estimation.

Our statistical analyses revealed a significant group main effect for the entrainment responses [*F*(1,45) = 49.96; *P* < 0.001], indicating the adults with DS had weaker neural activity in the occipital cortices compared to controls across all three harmonics (DS: 12.5 ± 7.3%, NT: 89.3 ± 7.9%). There was not a harmonic main effect (*P* = 0.176). However, there was a significant group by harmonic interaction [*F*(2,90) = 9.57; *P* < 0.001]. As shown in Fig. 3, the *post hoc* tests indicated that the adults with DS had weaker entrainment responses at 14–16 Hz (*P* < 0.001), 29–31 Hz (*P* < 0.001) and 44–46 Hz (*P* = 0.008) when compared with the controls, with the most robust differences being at the fundamental frequency (i.e. 14–16 Hz). Details on the descriptive statistics, *post hoc* tests and effect sizes are displayed in Table 1.

**Table 1:** Entrainment results

Frequency	Down syndrome mean ± SD (95% CI)	Neurotypical control mean ± SD (95% CI)	t-statistic	*P* value	Cohen’s *d*
14–16 Hz	9.1 ± 21.6%	116.1 ± 65.7 (0.4–17.8)	−7.54 (87–145.2)	<0.001	2.2
29–31 Hz	15.3 ± 16.1%	102.3 ± 82.6% (8.8–21.8)	−5.07 (65.7–139.0)	<0.001	1.80
44–46 Hz	10.4 ± 14.2%	52.7 ± 51.7% (4.6–16.1)	−3.73 (29.8–75.6)	0.008	0.82

Note that *P*-values were Bonferroni corrected. CI, confidence interval; SD, standard deviation.

**Figure 4:**
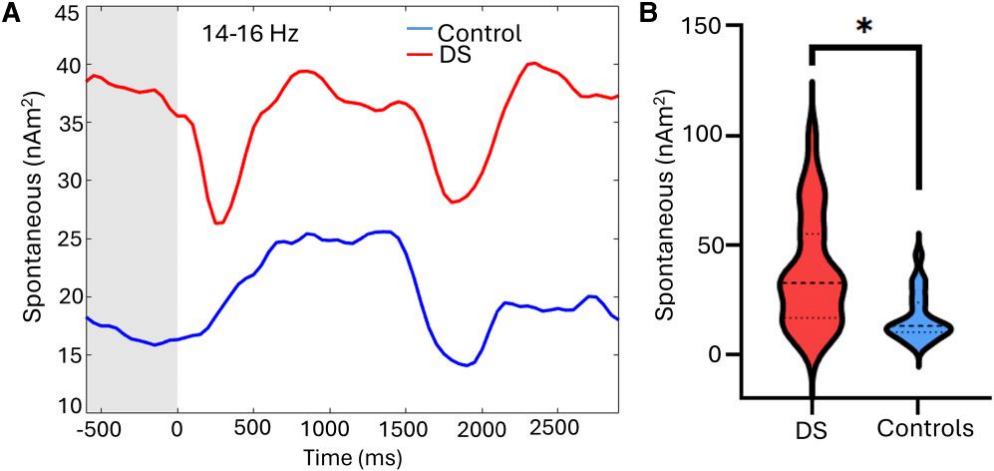
**Spontaneous 14–16 Hz activity in the occipital cortices during the baseline period.** The left panel displays the non-normalized (i.e. absolute) neural time series per group, with the blue line reflecting the adult neurotypical controls (*N* = 22) and the red line showing the adults with Down syndrome (DS; *N* = 26). Time 0 ms on the abscissa represents the onset of the 15 Hz visual stimulus, while the shaded area represents the baseline period from which the mean spontaneous activity was calculated. A repeated-measures ANOVA model (group × harmonic) with age as a covariate of no interest was used to evaluate differences in spontaneous activity during the baseline. As shown, spontaneous activity was significantly elevated in those with DS compared to the controls [*t*(45) = 3.66; *P* = 0.01]. Violin plots representing the average strength of spontaneous 14–16 Hz activity per group during the baseline period (−600 to 0 ms) is shown in the right panel. The respective group medians and quartiles are shown on the violin plots. *P = 0.05.

Regarding spontaneous activity, there was a significant group main effect [*F*(1,45) = 12.59; *P* < 0.001], showing that the strength of the spontaneous activity was overall stronger in the adults with DS compared with the controls (DS: 18.1 ± 1.86 nAm2; NT: 16.84 ± 10.72 nAm2). There was also a significant main effect of harmonic [*F*(2, 90) = 3.64; *P* = 0.03]. *Post hoc* tests showed that the spontaneous activity in the 14–16 Hz band (27.33 ± 2.95 nAm2) was significantly stronger than the 29–31 Hz (8.31 ± 0.98 nAm2; *P* < 0.001) and 44–46 Hz (3.88 ± 0.56 nAm2; *P* < 0.001) harmonics. Additionally, there was a significant group by harmonic interaction [*F*(2,90) = 12.6; *P* < 0.001]. As shown in Fig. 4, the *post hoc* tests revealed that the adults with DS had stronger spontaneous activity during the baseline at 14–16 Hz (*P* = 0.01), but not 29–31 Hz (*P* = 0.122) or 44–46 Hz (*P* = 1.0). Details on the descriptive statistics, *post hoc* tests, and effect sizes are displayed in Table 2.

**Table 2:** Spontaneous results

Frequency	Down syndrome mean ± SD (95% CI)	Neurotypical control mean ± SD (95% CI)	t-statistic	*P* value	Cohen’s *d*
14–16 Hz	37.91 ± 25.7 nAm^2^	16.8 + 10.7 nAm2 (27.51–48.3)	3.66 (12.09–21.59)	0.01	1.74
29–31 Hz	10.77 ± 9.1 nAm^2^	5.90 ± 2.6 nAm2 (7.07–14.6)	2.76 (4.73–7.07)	0.122	0.43
44–46 Hz	4.81 ± 5.0 nAm^2^	2.97 ± 1.7 nAm2 (2.78–6.83)	1.82 (2.19–3.75)	1.0	0.16

Note that *P*-values were Bonferroni corrected. CI, confidence interval; SD, standard deviation.

Finally, given the spectrogram data showing a response in adults with DS in the 8–12 Hz range, we conducted an exploratory analysis to examine whether this response differed by group. First, we imaged 8–12 Hz activity in the 200–400 ms time window shown in the time–frequency spectrogram. The beamformer revealed that the 8–12 Hz power decrease emanated from the bilateral occipital cortices, which is in agreement with prior literature showing a decrease in alpha power during the initial processing of visual stimuli.^36^ The neural time courses were subsequently extracted for each participant based on the peak voxel location. Statistical analyses revealed that the strength of this alpha response did statistically differ between controls and the adults with DS [*t*(46)= −1.175, *P* = 0.246]. Further details on these alpha responses can be found in the Supplementary Materials.

## Discussion

This investigation evaluated whether the entrainment of occipital cortical neurons and spontaneous rhythms within the same neural populations were aberrant in adults with DS. As hypothesized, our MEG results indicated that adults with DS exhibited significantly weaker entrainment compared with the controls. In addition, we found that the strength of spontaneous baseline-level activity in the occipital cortices was sharply elevated in adults with DS relative to matched controls. We discuss the implications of these novel findings in the following paragraphs.

One of our key findings was that the capacity of occipital cortical neurons to entrain to the 15 Hz flicker stimulation was far less in people with DS compared to controls. This finding complements the prior EEG literature, which has shown that children and adults with DS also display weaker visual-evoked potentials at the electrode level.^17-19^ In addition, these findings align with Ts65Dn animal models of DS that have shown reduced visual evoked potentials while viewing a wide array of stimuli.^37^ We suspect that the reduced entrainment observed here might align with the perspective that adults with DS have elevated GABA neurotransmitter concentrations at the synaptic level and/or a higher number of GABAergic interneurons.^38^ However, this perspective is not fully aligned with observations from post-mortem brain tissue, which have shown that GABA levels are reduced or unchanged in individuals with DS.^39,40^ In addition, prior magnetic resonance spectroscopic (MRS) studies have shown a reduction in GABA concentrations in persons with DS between the ages of 3–17 years,^41,42^ although these data were not from the occipital cortices and the sample sizes were limited.

It is alternatively plausible that the reduced entrainment was simply driven by a decrease in the number of pyramidal neurons in the occipital cortices. This speculation is partially supported by Ts65Dn animal models of DS, which have revealed that the genetic disturbance results in a reduction in the neuronal density, with pyramidal neurons that have fewer dendritic branches and synaptic spines.^43,44^ Evaluation of the morphology of post-mortem tissue from the occipital lobe of individuals with DS has also revealed the presence of shorter dendrites, decreased number of spines and a 50% reduction in the number of cells.^45,46^ That being said, the structural MRI outcomes from children and adults with DS have been mixed, with some studies reporting a reduction in the thickness of the occipital cortices,^9^ while others report an increase.^47,48^ Although again, sample sizes have tended to be on the smaller side. Bluntly, there is a critical need to isolate the precise mechanisms underlying the sharply reduced capacity for neuronal entrainment in the occipital cortices of adults with DS.

Our second major finding was the elevated spontaneous cortical activity observed in adults with DS. We suspect that this signifcantly stronger spontaneous activity played at least a partial role in the weaker entrainment seen in the adults with DS. In other words, entrainment to the visual flicker stimulus might have been weaker because the basal spontaneous activity was closer to the ceiling of the neural population’s capacity for synchronized disharges. Prior MEG experimental work has supported such dynamic range effects, as multiple studies have shown that increases in spontaneous cortical activity affects the capacity of local neural populations to generate oscillations at the same frequency.^49-60^ Notably, these prior studies have generally focused on broader bands (e.g. beta activity) and the increases in spontaneous activity were due to aging and/or neurological diseases. The limited data on 15 Hz spontaneous activity suggests that it does not differ as a function of age in the general populaton.^22^ As such, the heightened spontaneous cortical activity seen in this investigation might represent changes due to the altered genetics. Future work is needed to more fully understand the implications, especially given that the group differences in the current study were quite large in terms of effect size. Prior MEG and EEG investigations have also reported that spontaneous activity during the resting-state is elevated from the theta to alpha frequency ranges in the occipital cortices of persons with DS compared to controls.^16,61^ Thus, the elevated level of spontaneous activity that we report here appears to closely correspond to these prior findings. Together, these results suggest that there might be fundamental neurobiological changes in persons with DS that are responsible for the elevated spontaneous activity. Identifying the precise mechanism(s) underlying such aberrant spontaneous activity may illuminate thereputic targets for the treatment and/or prevention of dementia and other cognitive aberrations often seen in this patient population.

The motivation for this investigation was to begin working towards the identification of a neurophysiological marker that can be used for the early detection of dementia in adults with DS. Prior MEG research has shown that patients with AD tend to have stronger 15 Hz entrainment in the occipital cortices and similar spontaneous activity levels during the baseline period compared to controls.^21^ These findings are clearly different than what was observed here in adults with DS. Albeit this may be somewhat expected given that the inclusion criteria for this investigation was the absence of a dementia diagnosis. We suggest that if the neurophysiological profiles are similar between persons with AD and adults with DS that have dementia, then a marked increase in the strength of the occipital cortical entrainment might be the expected biomarker. Alternatively, it could be that the deviations in spontaneous cortical activity, as observed here, reflect genetically driven neurophysiological changes, which alter the entrainment pattern in ways that do not and will not align with the neurophysiology of AD. While possible, it could also be the case that the emergence of early stages of dementia leads to abrupt changes in spontaneous activity. To test this premise, a cohort of adults with DS and a diagnosis of dementia should be enrolled in future studies.

Before closing, it is important to note the limitations of this study. First, despite collecting comprehensive neurophysiological data, we did not perform comprehensive clinical tests. Future studies should consider integrating neuropsychological batteries to explore the functional impact of the alterations in cortical entrainment seen in individuals with DS. However, it is also well recognized that there are limitations in the accuracy of standardized tests used to quantify the extent of cognitive deficiencies seen in adults with DS,^62^ which makes the discovery of neural markers of cognitive dysfunction in DS even more important. Additionally, this study only utilized one entrainment frequency. A potentially fruitful future direction would be to evaluate occipital entrainment at the gamma frequency,^63^ as it is tightly coupled with GABAergic signalling.^64-67^ Essentially, this experiment would further probe the current hypothesis that adults with DS have heightened GABAergic inhibition.^38^ Alternatively, there is emerging evidence there might be an interplay between the degeneration of basal forebrain cholinergic neurons and the early onset of AD in individuals with DS.^68^ Potentially, the disruption in the cortical oscillations seen here might also be related to a disruption in the cholinergic neurons, as prior research as shown they play a role in visual perception.^6^ We propose that future studies using magnetic resonance spectroscopy and/or PET will provide direction in regard to potential neurochemical and/or cellular receptor deviations that may be potentially responsible for the altered visual processing reported in our investigation. Lastly, our study sample included adults with DS and no dementia diagnosis. In future studies, inclusion of individuals with DS from pre-clinical stages to more advanced stages of AD would be highly informative and a key next step to furthering our understanding of Alzheimer’s neuropathology in individuals with DS.

## Conclusions

The current study represents the first MEG investigation of cortical entrainment in adults with DS. Our results show that adults with DS exhibit prominently weaker entrainment of occipital cortical neurons and elevated spontaneous activity during the baseline period compared to controls. We suspect that these alterations might reflect aberrant GABAergic function, disruptions in the cholinergic pathways, and/or genetic influences on cortical dynamics. These unique results provide a new framework for understanding cortical processing deficits in adults with DS and may hold promise in the identification of biomarkers for the early detection of accelerated age-related cognitive decline in DS.

## Supplementary Material

fcag038_Supplementary_Data

## Data Availability

Data will be made available upon reasonable request.
