# Supplementary material for: Adults with Down syndrome display altered entrainment of occipital cortical neurons
Source: Brain Commun. 2026 Feb 6;8(1):fcag038. doi: 10.1093/braincomms/fcag038 (PMC12922442; doi:10.1093/braincomms/fcag038)
Supplement: fcag038_Supplementary_Data [file fcag038_supplementary_data.pdf]

Supplementary Material

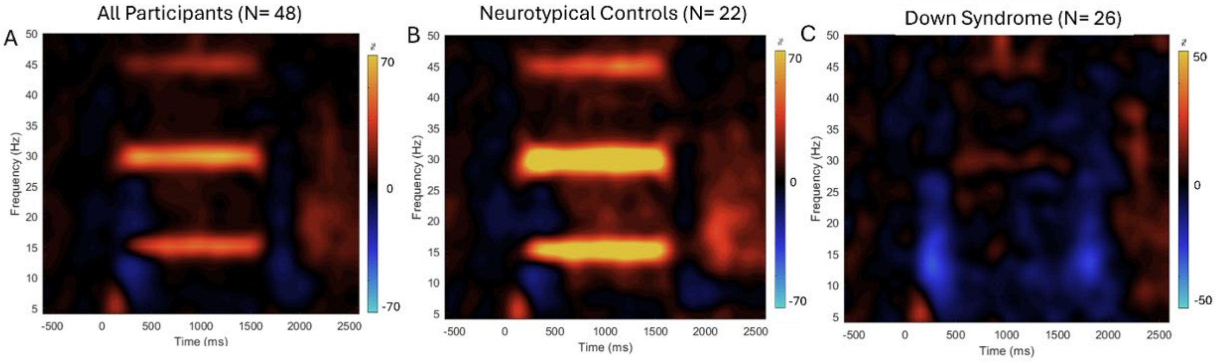

Figure S1: Color-blind friendly depiction of the sensor level neural responses. Group-averaged time frequency spectrograms for a sensor near the occipital cortices are shown where the x-axis depicts time (ms) and the y-axis corresponds to frequency. 0 ms is the onset of the 15 Hz flickering stimulus. The heat map scale bars represent the percent change from the baseline where a yellow color indicates power increase relative to the baseline, while a light blue represents a decrease. The respective panels show the spectrograms generated based on the data from all of the participants (A), only the neurotypical controls (N=22) (B), and the adults with Down syndrome (N=26). The grand averaged and control group spectrograms revealed that there was a robust power increase at 15 Hz that stretched across the 250-1600 ms time window, with strong responses also seen at the second and third harmonics (i.e., 30 and 45 Hz, respectively). However, this was not the case for the adults with Down syndrome, where entrainment to the 15 Hz stimulus was absent and responses at the second and third harmonics were very weak. Note that the scale of the spectrogram for the Down syndrome group is shown at a much more sensitive level than the grand-averaged and control spectrograms. This was necessary to illustrate the harmonic responses, although it also makes the power decrease in the 10-20 Hz range following stimulus onset and offset appear more strongly.

A beamformer was used to image the source of the 8-12 Hz power decrease that was seen within the 200-400 ms time window in each participant using a -200 to 0 ms baseline. The images revealed that the activity emanated bilaterally from the occipital cortices (Figure S2A). Neural time courses were subsequently extracted for each participant based on the peak voxel location seen in the respective hemispheres. These time courses were then averaged since there were no laterality hypotheses, and the mean response from 200-400 ms was calculated (Figure S2B). An independent t-test was used to assess if there were group differences. The statistical test showed that there was not

a significant difference between the controls and the adults with DS for the cortical response (Figure S2C; DS=-25.93 ±15.9%; NT= -20.66 ±14.9%; t(46)= -1.175, p = .246).

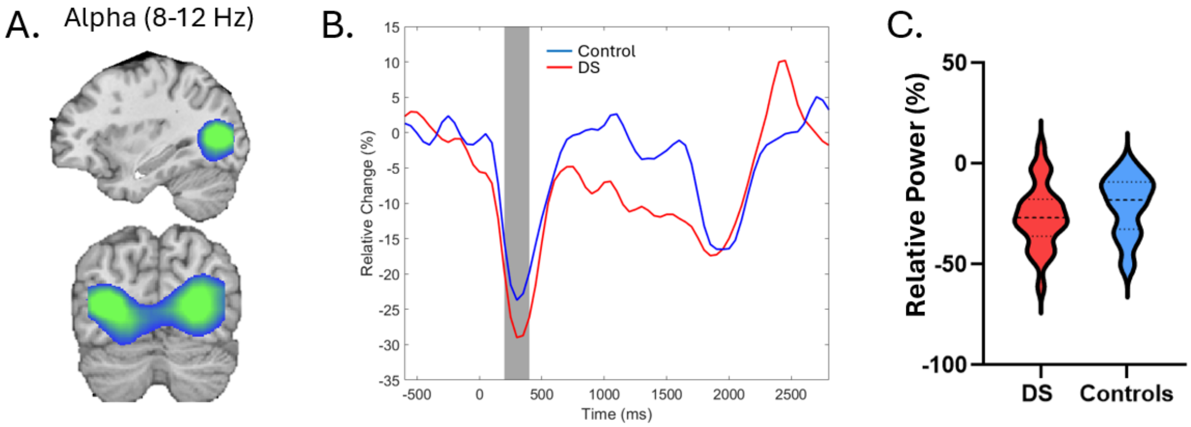

Figure S2. Alpha (8-12 Hz) cortical oscillations. A) The beamformer images show that the source of the alpha (8-12 Hz) cortical response was bilateral occipital cortices. B) The neural time courses were extracted from the peak voxel of the respective hemispheres and averaged since there were no laterality hypotheses. The blue line represents the neural time course for the neurotypical controls and the red line represents the adults with Down syndrome (DS). Time 0 ms reflects the onset of the 15 Hz visual stimulus, while the shaded area represents the time window used for imaging. C) The violin plots display the average strength of the alpha power decrease within the 200 to 400 ms time window per group. The median (50th percentile) is indicated by the dashed line and the quartiles (25th and 75th) are marked by the dotted lines. An independent t-test revealed that there was not a significant difference in the strength of the alpha oscillations (*p* = .246). Green colors indicate higher activation, while blue colors indicate a lower activation.
